# Sigma-2 Receptors—From Basic Biology to Therapeutic Target: A Focus on Age-Related Degenerative Diseases

**DOI:** 10.3390/ijms24076251

**Published:** 2023-03-26

**Authors:** Britney N. Lizama, Jennifer Kahle, Susan M. Catalano, Anthony O. Caggiano, Michael Grundman, Mary E. Hamby

**Affiliations:** 1 Cognition Therapeutics, Inc., Pittsburgh, PA 15203, USA; 2 IHS International, San Diego, CA92130, USA; 3 Global R&D Partners, LLC., San Diego, CA 92130, USA; 4 Department of Neurosciences, University of California, San Diego, CA 92093, USA

**Keywords:** S2R, TMEM97, PGRMC1, MAC30, σ2R, Alzheimer’s disease (AD), Parkinson’s disease (PD), prion protein, PrP^C^, dry age-related macular degeneration (dry AMD), dementia with Lewy bodies (DLB), Niemann–Pick disease type C (NPC), schizophrenia, Huntington’s disease, α-synuclein, degenerative disease

## Abstract

There is a large unmet medical need to develop disease-modifying treatment options for individuals with age-related degenerative diseases of the central nervous system. The sigma-2 receptor (S2R), encoded by *TMEM97*, is expressed in brain and retinal cells, and regulates cell functions via its co-receptor progesterone receptor membrane component 1 (PGRMC1), and through other protein–protein interactions. Studies describing functions of S2R involve the manipulation of expression or pharmacological modulation using exogenous small-molecule ligands. These studies demonstrate that S2R modulates key pathways involved in age-related diseases including autophagy, trafficking, oxidative stress, and amyloid-β and α-synuclein toxicity. Furthermore, S2R modulation can ameliorate functional deficits in cell-based and animal models of disease. This review summarizes the current evidence-based understanding of S2R biology and function, and its potential as a therapeutic target for age-related degenerative diseases of the central nervous system, including Alzheimer’s disease, α-synucleinopathies, and dry age-related macular degeneration.

## 1. Introduction

The leading causes of memory loss, cognitive decline, and blindness are age-related degenerative diseases of the central nervous system. Age-related degenerative diseases are defined by an age-associated degeneration of cellular function and cell death, in which toxic proteins, oxidative stress, inflammation, and pathophysiological cell signaling are present and contributing factors to disease progression. Despite the progress in understanding the pathology of these diseases, there are limited treatment options for these diseases, which present with a decline in function, including motor, sensory, and cognitive capabilities, and result in decreased quality of life and shorter life span. While there are several proteins and pathways implicated in contributing to age-related diseases, the sigma-2 receptor (S2R) acts as a regulator of cellular damage associated with certain age-related degenerative diseases of the central nervous system, including Alzheimer’s disease, Parkinson’s disease, dementia with Lewy bodies, and dry age-related macular degeneration (dry AMD).

S2R is a four-domain transmembrane receptor, also known as transmembrane protein 97 (TMEM97), that is expressed in multiple cell types susceptible to degeneration, including neurons [1], and regulates cell functions disrupted in disease, including cholesterol biosynthesis/trafficking, membrane trafficking, autophagy, lipid membrane-bound protein trafficking, and receptor stabilization at the cell surface. The aberrant activity of these processes, believed to be triggered by cellular stresses, is another common feature in age-related degenerative diseases. Modulation of S2R may ameliorate dysfunctional cellular processes and slow degenerative progression. Preclinical studies support that S2R small molecule modulators can rescue biological processes that are impaired in neurodegenerative diseases [2,3,4,5]. While there are several synthetic ligands for S2R, classifying these ligands as “agonist/antagonist” may be inaccurate, given that functional activity may be context dependent. S2R ligands are often undercharacterized across a host of distinct functional assays, and agonist/antagonist nomenclature is usually ascribed based on a functional phenotype. Still, the array of degenerative diseases that involve S2R and interacting proteins supports the further development for therapeutic application of S2R modulators in a wide range of age-related degenerative diseases. Targeting S2R represents a novel approach that is functionally distinct from other current approaches in the development of clinical treatments of degenerative diseases.

Interest in S2R has grown given its high expression in the brain, retina, and other organs, as well as its role in diverse physiological processes and diseases throughout the body [1,6]. There are several recent reviews of S2R and closely associated proteins in many of these roles and contexts, including reproduction [7,8,9], cancer [10,11], Alzheimer’s disease [12,13,14], nerve growth [14,15], molecular functions [16], cytochrome P450 interactions [17], and lipid metabolism [18]. This review will focus on summarizing the current evidence-based understanding of S2R and its potential as a therapeutic target for age-related degenerative diseases of the central nervous system. The aberrant activity of S2R-mediated cellular processes, believed to be triggered by cellular stresses and exacerbated in aged cells, is a hallmark of the dysfunction underlying degenerative diseases, including Alzheimer’s disease, α-synucleinopathies, such as Parkinson’s disease and dementia with Lewy bodies, and retinal diseases, such as dry AMD.

## 2. Alzheimer’s Disease

### 2.1. Overview of the Disease

Alzheimer’s disease is a progressive neurodegenerative disorder characterized by several pathological hallmarks, including amyloid-β pathology, tau pathology (i.e., neurofibrillary tangles), and inflammation, in which individuals present with cognitive dysfunction, memory loss, dementia, and the impairment of daily living activities [19]. In the advanced stages of the disease, individuals with Alzheimer’s disease are unable to recognize faces, unable to use or understand language, and display a lack of awareness for their surroundings. Continued functional decline ultimately results in the individual’s death. Due to the size of the affected population and the current lack of effective disease-modifying therapies, Alzheimer’s disease is one of the most serious unmet medical needs of our time. Nearly six million people in the United States alone have been diagnosed with Alzheimer’s disease and disease prevalence is expected to more than double by 2050 [19]. The direct healthcare costs to care for individuals with Alzheimer’s disease and other dementias in the United States is currently estimated to exceed USD 300 billion and projected to increase to USD 1 trillion by 2050 [19]. Absent is the development of meaningful interventions in the course of the disease, as the number of people suffering with Alzheimer’s disease is anticipated to escalate appreciably as lifespans lengthen, because prevalence increases significantly with age. The Centers for Disease Control listed Alzheimer’s disease as the primary cause of death for more than 121,000 Americans in 2019. The disease is equally devastating worldwide, with the World Health Organization estimating that Alzheimer’s disease affects as many as 35 million people globally [20].

### 2.2. Currently Approved Alzheimer’s Disease Therapeutics

Most therapies approved for Alzheimer’s disease are indicated to only treat the symptoms of Alzheimer’s disease: acetylcholinesterase inhibitors, a glutamatergic modulator (memantine), and an orexin receptor antagonist (suvorexant) for Alzheimer-related insomnia [21,22]. Acetylcholinesterase inhibitors slow the degradation of the neurotransmitter acetylcholine, helping to preserve neuronal communication and function. Glutamatergic modulators block sustained, low-level activation of the *N*-methyl-d-aspartate receptor without inhibiting the normal function of the receptor in memory and cognition [23]. Memantine was approved in the United States in 2003. It is used to help support cognitive function until late in disease progression [24]. Suvorexant is a dual orexin receptor antagonist, used for treatment of insomnia, which may exacerbate symptoms of dementia in patients with Alzheimer’s disease [22]. The medications noted above do not alter the progression of the underlying disease and provide only modest efficacy in treating symptoms.

Two anti-amyloid-β monoclonal antibodies have been approved by the Food and Drug Administration (FDA) for the treatment of early-stage Alzheimer’s disease. Specifically, Biogen’s aducanumab received accelerated approval on 7 June 2021 and Eisai’s lecanemab received accelerated approval on 6 January 2023 based on the observation of amyloid plaque removal (a surrogate endpoint) observed in individuals with Alzheimer’s disease treated with these agents. Continued approval of these medications, however, is dependent upon verification of clinical benefit in confirmatory trials, or following review of the full clinical data package from completed clinical trials.

The Phase 3 clinical trial evaluating lecanemab (an anti-amyloid-β monoclonal antibody that targets protofibrillar and oligomeric forms of amyloid-β) in individuals with early Alzheimer’s disease was recently published and indicated that lecanemab met the primary endpoint of the trial of slowing clinical decline by 27% on the Clinical Dementia Rating Sum of Boxes. Additionally, like aducanumab, it also reduced amyloid-β plaques in the brains of individuals with Alzheimer’s disease [25]. The effect of lecanemab on slowing cognitive and functional decline was associated with infusion related reactions and the development of amyloid-related imaging abnormalities with edema (ARIA-E) or effusions in 12.6% of individuals. Similarly, ARIA-E occurred in 35.2% of individuals treated with aducanumab in Phase 3 clinical trials [26]. Given the ARIA-E observed with monoclonal antibody approaches, it will be important for the field to continue to pursue the development of alternative therapeutic modalities and approaches for Alzheimer’s disease that do not present with ARIA-E, for example, small molecules approaches.

### 2.3. Therapeutic Approaches in Development to Treat the Underlying Disease Have Shown Modest Success

Numerous therapeutic approaches targeting tau, neuroinflammation, and other mechanisms to remedy the causes of Alzheimer’s disease have been evaluated or are currently being evaluated in clinical trials [27], with the majority of attempts to bring efficacious therapies to patients resulting in failure [28]. Approaches targeting tau focused on reducing the aberrant production, or removal, of intraneuronal neurofibrillary tangles of tau protein have yielded limited clinical benefit [29]. Development initiatives intended to inhibit hyperphosphorylation of the tau protein and related kinase activity, enhance microtubule stability, or block tau aggregation have largely been discontinued due to toxicity or a lack of efficacy. Microglial activation and its role in Alzheimer-induced neuroinflammation has emerged as another potential target for therapeutic development as has the proper functioning of processes dictating synaptic plasticity, believed to be of central importance to neuronal activity and continued viability. Ongoing clinical trials are testing the hypothesis that specific modulation of inflammation will prove beneficial to individuals with Alzheimer’s disease, including TREM2 monoclonal antibody approaches (Alector, AL002 NCT04592874 A Phase 2 Study to Evaluate Efficacy and Safety of AL002 in Participants With Early Alzheimer’s Disease (INVOKE-2)).

Among the more prevalent and targeted mechanisms implicated in Alzheimer’s disease is the accumulation of amyloid-β aggregates in the neuronal synapse, where disease progression leads to synaptic dysfunction and dysregulation [30]. The accompanying deterioration in neuronal activity ultimately results in neuronal death. The reduction in the levels of amyloid-β plaques in the brain has been a prominent objective of a significant number of therapeutic candidates, including active and passive immunotherapies, designed specifically to target amyloid-β aggregates. Many amyloid-β lowering approaches have failed [31]. A potential weakness of current therapeutic interventions intended to reduce amyloid-β plaques in the brain is that although these approaches may clear amyloid fibrils and the largely inert plaques, they may only partially address the specific neurotoxic effects of amyloid-β oligomers [30], which are suggested to be the most neurotoxic forms of amyloid protein implicated in the neurodegeneration observed in Alzheimer’s disease [32]. The clinical benefit observed in trials with lecanemab, which targets protofibrils and oligomers [25], provides encouragement for pursuing additional therapies targeting amyloid-β oligomers. A novel small molecule approach targeting S2R with the potential to prevent amyloid-β oligomer toxicity by acting indirectly on oligomers at the level of the synapse is currently being tested in the clinic (NCT03507790, NCT04735536). There may be potential synergies between the S2R modulator CT1812, which displaces amyloid-β oligomers from synapses (discussed further below), and anti-amyloid-β monoclonal antibodies that may bind to and increase the clearance of these displaced amyloid-β oligomers.

## 3. α-Synucleinopathies—Parkinson’s Disease and Dementia with Lewy Bodies

### 3.1. Overview of the Disease

α-Synucleinopathies are a group of neurodegenerative disorders in which the protein α-synuclein accumulates abnormally to form inclusions in the cell bodies or axons of neurons or oligodendrocytes. Two of the primary α-synucleinopathies are Parkinson’s disease and dementia with Lewy bodies, also known as Lewy body dementia, both resulting in motor and cognitive dysfunction. While the cell types and brain structures that are affected in Parkinson’s disease and dementia with Lewy bodies differ between the disorders, α-synucleinopathies share a characteristic accumulation of α-synuclein aggregates into fibrils, the major constituent of the Lewy bodies that occur inside brain neurons in both diseases.

Increasing evidence suggests that α-synuclein also forms soluble oligomers, and that oligomers are more toxic than fibrils [33,34,35]. α-Synuclein oligomers contribute to neurodegeneration through a variety of mechanisms including disrupting normal autophagy and inducing synaptic dysfunction and loss [36,37]. Synaptic dysfunction and loss contribute to the cognitive and motor symptoms of these diseases.

α-Synucleinopathies are second only to Alzheimer’s disease in terms of neurodegenerative disease prevalence. In the United States, as many as one million people suffer from Parkinson’s disease and an estimated 1.4 million from dementia with Lewy bodies, approximately 5–10% of all dementia cases [38]. According to the Parkinson’s Foundation and the Lewy Body Dementia Association, the direct healthcare costs for individuals with Parkinson’s disease and dementia with Lewy bodies are estimated to be approximately USD 25 billion and USD 31.5 billion per year, respectively [39]. For Parkinson’s disease, these direct medical costs include an estimated USD 2.5 billion for medications annually in the United States.

Dementia with Lewy bodies has overlapping pathology and symptomology with Parkinson’s and Alzheimer’s diseases, making it challenging to diagnose. Individuals with dementia with Lewy bodies often experience cognitive, physical, sleep, and behavioral symptoms, including hallucinations, delusions, and mood changes. There are currently no disease-modifying treatments approved for individuals with dementia with Lewy bodies.

### 3.2. Limitations of Current Treatments for α-Synucleinopathies

Therapeutic products for these diseases are directed toward symptomatic relief. Some dopamine enhancing type treatments have been approved for Parkinson’s disease (e.g., l-DOPA and dopamine receptor agonists) [40]; however, no specific therapeutic agents have received regulatory approval for treatment of dementia with Lewy bodies. While some existing therapeutics provide meaningful symptomatic benefits, they often have significant side effects and, over time, gradually lose their effectiveness [40]. There are no currently approved disease-modifying therapeutics for Parkinson’s disease, dementia with Lewy bodies, or other α-synucleinopathies. Multiple therapeutic approaches are being pursued targeting α-synuclein, oxidative stress, mitochondria dysfunction, and other mechanisms, which have been reviewed elsewhere [41]. The S2R is a therapeutic target under investigation for α-synucleinopathies and CT1812 is currently in Phase 2 trials, being developed by Cognition Therapeutics, for dementia with Lewy bodies (NCT05225415). Support for targeting the S2R derives from α-synuclein binding to PrP^c^ [42], and the finding that S2R modulators block α-synuclein oligomer toxicity in neurons [4]; this is a topic discussed in greater detail below.

## 4. Retinal Diseases—Dry Age-Related Macular Degeneration

### 4.1. Overview of the Disease

AMD is the leading cause of blindness in people over 50 years of age in the United States, afflicting approximately 11 million people in the United States, including an estimated 12% of all American adults over 80 years of age, for which there are no disease-modifying therapies approved [43]. There are two types of AMD, the first of which is neovascular, or wet AMD, and non-neovascular, or dry AMD. Dry AMD is a progressive condition and accounts for the majority of all AMD cases [43]. Advanced dry AMD affects approximately 2 million people in the United States [44]. Dry AMD involves a dysregulation of cellular processes, among which is the accumulation of lipid deposits, known as drusen, that causes a thickening of Bruch’s membrane [45]. This thickening disrupts the cytoarchitecture of the retinal pigment epithelium, and this disruption, coupled with oxidative stress and inflammation, leads to the diminished health and function of retinal pigment epithelial and photoreceptor cells, with accumulated damage resulting in cell death and visual impairment.

### 4.2. Treatments for Dry AMD Are Limited

Treatments for dry AMD are currently limited to vitamins and over-the-counter zinc [46]. While there are no therapeutics approved by the FDA to treat dry AMD, there is considerable ongoing development involving numerous targets. Among the areas of ongoing interest are efforts targeting the complement pathway and its role in inflammation, as mutations in this pathway have been associated with higher risk of dry AMD [47]. Two new treatments, both of which are injected directly, intravitreously, into the eye, have recently completed testing in late-stage clinical trials, both targeting inhibition of the complement pathway; pegcetacoplan (APL-2), being developed by Apellis, targets a complement protein called C3, and avacincaptad pegol, developed by Iveric, targets C5. The GATHER2 Phase 3 clinical trial of Zimura met its primary endpoint showing a 14.3% reduction (*p* = 0.0064) in mean rate of growth in the geographic atrophy area over months [48].

Pegcetacoplan, under the name Syfovre, was very recently approved by the FDA in February 2023 as the first treatment for geographic atrophy secondary to dry AMD, and is expected to enter the market in 2023 [49,50]. The approval was based on positive 24-month results from Apellis’ late-stage Derby and Oaks studies, which both showed robust and consistent decreases in the change in geographic atrophy lesion size from baseline with pegcetacoplan treatment. While this is an exciting advance for the field, this is not a cure, and there remains a strong need to bring forward additional therapeutics for dry AMD [50].

Other approaches being pursued include invasive cell and gene therapy approaches to regenerate retinal pigment epithelial cells and rescue the loss of photoreceptors, and small molecule visual cycle modulators to maintain retinal integrity. Given that most of the treatments in development are invasive in nature [45], an orally delivered small-molecule approach targeting S2R would offer several advantages over these approaches and/or may be a complementary approach in combination with invasive therapeutics if proven efficacious in clinical trials. A Phase 2 clinical trial for dry AMD testing the ability of S2R modulators to slow disease progression is currently planned by Cognition Therapeutics.

## 5. The Sigma-2 Receptor

### 5.1. History, Structure, and Function of S2R

**Figure 1:**
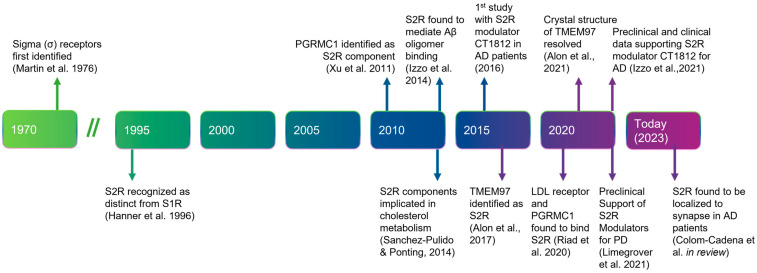
Timeline of the discovery and elucidation of S2R from inception to therapeutic modulation. References: Martin et al., 1976 [57]; Hanner et al., 1996 [58]; Xu et al., 2011 [59], Izzo et al., 2014 [2]; Sanchez-Pulido & Ponting, 2014 [60]; Alon et al., 2017 [61]; Riad et al., 2020 [62]; Alon et al., 2021 [63]; Izzo et al., 2021 [64]; Limegrover et al., 2021 [4]; Colom-Cadena et al., 2021 [65]. Abbreviations: AD, Alzheimer’s disease; Aβ, amyloid-β; LDL, low-density lipoprotein; PD, Parkinson’s disease; PGRMC1, progesterone receptor membrane component 1; S1R, sigma-1 receptor; S2R, sigma-2 receptor; TMEM97, transmembrane protein 97.

Before describing the role of S2R in degenerative diseases in detail, we next summarize the history, structure, and function of S2R (Figure 1). A clear understanding of the structure and functionality of S2R has historically been elusive. S2R was first coined meningioma-associated protein 30 (MAC30). S2R activity was initially localized to a ~20 kDa membrane protein [51,52] and first thought to be progesterone receptor membrane component 1 (PGRMC1), based on mass spectrophotometry identification following an immunoaffinity pull down with an irreversible S2R ligand binder WC-11, physiochemical properties, and pharmacological evidence [53,54]. In later studies by two independent groups, however, in which PGRMC1 levels were genetically manipulated through overexpression or depletion, S2R binding activity was not altered irrespective of the level of PGRMC1 expressed, as assessed using the canonical S2R ligand tritiated di-o-tolylguanidine binding in the presence of saturating (+)-pentazocine, in a human epithelial cell line or a mouse motor neuron cell line [55,56]. Moreover, full activity persisted, even in the absence of PGRMC1.

**Figure 2:**
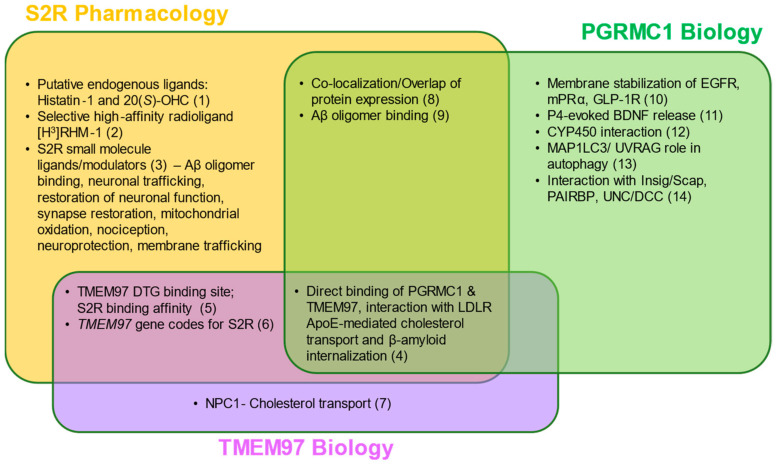
S2R pharmacology and the overlap with TMEM97 and PGRMC1 biology. Two putative endogenous ligands for S2R have been identified, although additional studies are needed to elucidate their roles under physiological and pathophysiological contexts. Despite this, several synthetic S2R ligands have been developed and used to investigate S2R function. Direct protein–protein interactions and functions of TMEM97 remain to be fully characterized; however, TMEM97 interacts directly with Niemann–Pick protein C1, as well as PGRMC1 and LDLR. PGRMC1 is more well-identified as a hormone receptor with multiple functions, including regulation of signal transduction pathways, protein–protein interactions, membrane trafficking, and autophagy. While ongoing studies attempt to elucidate the functions of each protein, S2R, TMEM97, and PGRMC1 converge in co-localization/co-expression and their regulation of amyloid-β binding. References: (1) [80,81]; (2) [82]; (3) [5,63,67,73,83,84,85,86,87,88,89,90]; (4) [62,66]; (5) [51,91,92]; (6) [16,61]; (7) [61,93,94]; (8) [1,59,95]; (9) [2,3,96]; (10) [97,98,99]; (11) [100]; (12) [78]; (13) [101,102]; (14) [103,104,105,106] Abbreviations: Aβ, amyloid-β; Apo-E, apolipoprotein-E; BDNF, brain-derived neurotrophic factor; DTG, di-o-tolylguanidine; EGFR, epidermal growth factor receptor; GLP-1R, glucagon-like peptide-1 receptor; Insig/Scap, insulin-induced gene/sterol regulatory element-binding protein cleavage-activating protein; LDLR, low-density lipoprotein receptor; MAP1LC3/UVRAG, microtubule-associated proteins 1A/1B light chain 3/UV radiation resistance associated gene protein; mPRα, membrane progesterone receptor alpha; NPC1, Niemann–Pick protein C1; PAIRBP, plasminogen activator inhibitor 1 mRNA-binding protein; PGRMC1, progesterone receptor membrane component 1; S2R, sigma-2 receptor; TMEM97, transmembrane protein 97; UNC/DCC, UNC-40/Deleted in Colorectal Cancer.

In 2017, the S2R was cloned [61], demonstrating that *TMEM97*, not *PGRMC1,* was the gene encoding S2R, indicating that S2R and TMEM97 are one and the same. This was confirmed in pharmacological studies assessing the activity of S2R ligands where reduction, or ablation, of S2R activity was observed when TMEM97 expression was reduced, or absent, in calf liver cells [16,61] or human cervical cancer cells [66], respectively. In further support, co-localization of TMEM97 with S2R ligands was demonstrated [67]. The identification of S2R as TMEM97 enabled a rapid advancement to the field, because it immediately merged two previously distinctly defined fields of research, enabling the application of that known about S2R pharmacology to TMEM97, and that known about TMEM97 biology to S2R (Figure 2) [61]. The crystal structure of TMEM97, although bovine, was resolved in 2021 which illuminated the docking sites of small molecule S2R ligands, PB28, and roluperidone [63], and allowed for structural enablement and docking for future medicinal chemistry efforts to identify S2R modulators that may be therapeutic candidates. This crystal structure also added additional confirmation that S2R is structurally distinct from the sigma-1 receptor [16,61,68].

### 5.2. Expression and Regulation of S2R/TMEM97

Within the brain, S2R is found in several areas, including the cerebellum, cortex, hippocampus, and substantia nigra, and is enriched in neurons as compared with glial cells in the adult brain [1]. Consistent with a role of S2R in synapses and in Alzheimer’s disease [69], S2R (TMEM97) has been demonstrated using Förster resonance energy transfer to colocalize with amyloid-β in synaptic densities [65]. In the retina, S2R is expressed in several cell types including the retinal pigment epithelium cells, photoreceptors, and retinal ganglion cells [70,71,72,73,74].

TMEM97 is a member of the EXPERA (EXPanded EBP superfamily) family of proteins along with emopamil binding protein (EBP), which is a protein domain containing four transmembrane regions and thought to have sterol isomerase activity [60]. Although TMEM97, unlike emopamil binding protein, is not thought to have isomerase activity, TMEM97 levels can be regulated by changes in cholesterol levels. More specifically, when cholesterol levels are reduced, TMEM97 expression can be induced by sterol regulatory element-binding protein-2 [75,76,77,78]. Both TMEM97 and PGRMC1 were regulated at the transcript level in skin cells by the topically applied Wnt/beta-catenin inhibitor C-82 under investigation in a clinical trial in individuals with systemic sclerosis, suggesting that Wnt signaling regulates TMEM97 [79]. Interestingly, increased expression of TMEM97 correlated with a rise in expression of genes involved in lipid metabolism, and these genes were significantly associated with the gene ontology terms cellular lipid, isoprenoid, cholesterol, and long-chain fatty-acyl-CoA biosynthetic processes [79], supporting a role of S2R in cholesterol biology. A further understanding of how TMEM97 may be regulated by different mediators and disease-relevant stressors is needed; however, in the case of Alzheimer’s disease, at least two reports suggest that TMEM97 is upregulated in synapses [65,69].

### 5.3. Putatitve Endogenous Ligands

Until recently, the endogenous ligand of S2R was unknown, although some ligands had been historically proposed but lacked conclusive substantiation in follow-on studies [107,108]. In 2021, two studies were published that identified two putative endogenous ligands of S2R: histatin-1 [80] and the oxysterol 20(*S*)-OHC [81]. Histatin-1 is a salivary protein with antibacterial and antifungal activities and is known to be involved in wound healing [109,110]. Histatin-1 is also found in human tears [111], and the functional binding of histatin-1 to S2R was confirmed in human corneal epithelial cells [80]. Because histatin-1 expression is highly enriched in, and fairly limited to, salivary glands [1,6] and tears, further research is needed to clarify if histatin-1 is a ligand in the majority of tissues where S2R is expressed, including the brain and retina [81]. In contrast, the biology of 20(*S*)-OHC alone makes it a notable candidate. First, 20(*S*)-OHC is involved in Niemann–Pick C1 (NPC1)-mediated cholesterol homeostasis, including lipid membrane and sphingolipid metabolism [81]. Further, it is known that TMEM97 and NPC1 physically interact [94] and oxysterols are known to post-translationally enhance the activity of enzymes involved in sphingomyelin biosynthesis [112]. The ligand binding site described for 20(*S*)-OHC in this model is remarkably consistent with the ligand-bound crystal structure of S2R, recently reported for S2R in complex with synthetic S2R ligands [63]. The resolution of the crystal structure of the ligand-bound S2R will further enable the discovery of additional endogenous ligands [113]. While researchers in the field continue to investigate the endogenous ligand for S2R, there remains a clear need to elucidate definitively what, if any, previously identified putative ligands have a role in S2R signaling under both physiological and pathophysiological settings.

### 5.4. Protein Interactions Underlie the Functions of S2R

**Figure 3:**
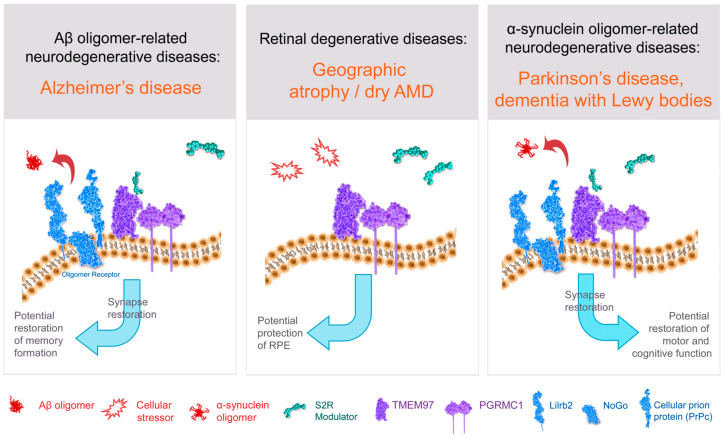
Structure–function of S2R and role of S2R small molecule modulators in restoring cell health and function. The amyloid-β oligomer receptor is a protein complex (light blue) that binds amyloid-β oligomers and is comprised of cellular prion protein (PrP^c^) along with other proteins including leukocyte immunoglobulin-like receptor subfamily B2/paired immunoglobulin-like type 2 receptor B (LilrB2), and possibly also Nogo-66 receptor 1 (Nogo). S2R small molecule modulator (green) binds to the S2R (TMEM97; purple). Abbreviations: AMD, age-related macular degeneration; Aβ, amyloid-β; PGRMC1, progesterone receptor membrane component 1; RPE, retinal pigment epithelium; S2R, sigma-2 receptor; TMEM97, transmembrane protein 97.

S2R couples and interacts with surrounding proteins to actuate a wide variety of cellular processes (Figure 3, Table 1). S2R has been shown to be closely associated and interact with key proteins to exert its functions, including PGRMC1 and LDLR [65,66]. PGRMC1 is a well identified hormone receptor with multiple functions in Alzheimer’s disease [114], α-synucleinopathies [4,115], and retinal disease [116]. Moreover, PGRMC1 has been speculated to be an adaptor protein involved in regulating intracellular signal transduction and/or membrane trafficking [11,117] given its immunoreceptor tyrosine-based activation motif sequence [106,117], which enables membrane trafficking as well as protein–protein interaction [11,117]. Indeed, S2R modulators have been shown to ameliorate amyloid-β oligomer and α-synuclein oligomer-mediated deficits in neuronal trafficking [2,3,4], a topic discussed further below.

Studies from multiple in vitro models have identified that both TMEM97 and PGRMC1 modulate autophagy, a quintessential cellular process needed to recycle cellular debris. PGRMC1 is a binding partner for numerous proteins in the autophagic pathway, including the key autophagic protein microtubule-associated protein 1A/1B light chain 3B (LC3) [101,102]. Treatment with S2R ligands induces processing of LC3, formation of autophagic vacuoles, and expression of downstream effectors of the mammalian Target of Rapamycin (mTOR) pathway [118]. *TMEM97* knockout cell lines exhibit aberrant autophagic flux and lysosome dysfunction [119]. Some small molecule modulators of S2R, such as siramesine and SV119, trigger the induction of autophagy [118,120], while other S2R modulators such as CT2168 can restore α-synuclein oligomer-induced deficits in lysosomal-associated membrane protein 2A levels, an indirect marker of autophagy, back to healthy control levels in neurons [4]. Given that autophagy is dysregulated in neurodegenerative conditions, further elucidation of S2R in regulating autophagy in brain and retinal disease is warranted, and may foster better translation into potential neuroprotective therapies.

One important function of S2R is regulation of cholesterol homeostasis by interacting with low-density lipoprotein receptor (LDLR), an apolipoprotein-E receptor, at the plasma membrane to transport apolipoprotein E into neurons [66] and with NPC1, intracellularly, to regulate LDL cholesterol transport out of lysosomes [93,94]. Cholesterol is a key component of the cell membrane and constitutes up to 30% of total membrane lipids [121]. Cholesterol, bound to LDL, is taken up into cells via the LDLR, and endocytosed. S2R is key in facilitating the uptake of LDL, as uptake of radiolabeled LDL was significantly decreased in *PGRMC1* knockout, *TMEM97* knockout, and double knockout cell lines [66,93]. Using these knockout cell lines, the same group demonstrated that both *PGRMC1* and *TMEM97* must be present and functional for effective LDL–LDLR complex internalization [66]. Further, to understand whether S2R alters all clathrin-mediated endocytic pathways or just the update of LDL, radiolabeled [^125^I]TYR^11^-somatostatin and [^125^I] insulin uptake was assessed, but knockout of *TMEM97* or *PGRMC1* did not alter the uptake of either substrate, indicating a specific role of S2R regulation of trafficking through LDLR [62].

The TMEM97/PGRMC1/LDLR triad is responsible for the uptake of amyloid-β oligomers and apolipoprotein E into neurons, as knockdown of TMEM97 or modulation of TMEM97 with S2R ligands including RHM-4 and SW43 substantially decreased the uptake of both amyloid-β oligomers and apolipoprotein E, but apolipoprotein E was not required for amyloid-β oligomer uptake [62]. The lack of dependence on apolipoprotein E for synaptic uptake has been reported and discussed previously [122,123]. Apolipoprotein E also interacts with PGRMC1 in the clearance of amyloid-β from neurons. Blocking the interaction between apolipoprotein E and PGRMC1 increases amyloid-β clearance from the brain, reduces amyloid-β deposition, and reduces related pathology in Alzheimer animal models [124]. This is consistent with results showing that specific small-molecule ligands that bind to S2R displace endogenous β-amyloid oligomers from human brain samples from individuals with Alzheimer’s disease, displace β-amyloid oligomer binding to primary cultured neurons, and reverse cognitive deficits in Alzheimer model mice [2,3].

There is a host of direct and indirect evidence linking S2R to the amyloid-β oligomer receptor. Amyloid-β oligomers bind to a protein complex comprised of cellular prion protein (PrP^c^) along with other proteins including leukocyte immunoglobulin-like receptor subfamily B2/paired immunoglobulin-like type 2 receptor B (LilrB2), and possibly also Nogo-66 receptor 1 (Nogo-1), low-affinity nerve growth factor (NGF) receptor [125] (Figure 3), and the metabotropic glutamate receptor 5 [126]. In the brain, the expressions of oligomer receptor constituents are enriched in neuronal synapses [126]. Several lines of evidence indicate a physical and/or functional interaction of S2R with this protein complex that binds amyloid-β oligomers. Amyloid-β oligomer binding results in aberrant neuronal signaling and the endocytosis of the dysfunctional synapse into the cell [126,127]. Amyloid-β oligomer binding to neuronal synapses is abrogated by knockdown of *PGRMC1* [3], PrP^c^, and with S2R modulators in neurons [2,3], an effect that leads to restoration of neuronal health and function (to be discussed in more detail below). Furthermore, treatment with a metabotropic glutamate receptor 5 modulator, which displaces amyloid-β oligomers, effectively prevented synaptotoxicity and restored functional synapses in a mouse model of Alzheimer’s disease [127]. Conditional deletion of the oligomer receptor PrP^c^ in Alzheimer transgenic mice rescues cognitive and synaptic deficits [128]. Lastly, to be discussed in greater detail below, S2R modulators restore cognitive deficits in Alzheimer transgenic mice [2,64]. In sum, substantial evidence linking S2R biology to that of the protein complex that binds amyloid-β oligomers supports the pursuit of small-molecule modulators of S2R to prevent amyloid-β oligomer-mediated toxicity.

## 6. S2R Modulators as a Promising Therapeutic Approach for Age-Related Degenerative Diseases

### 6.1. S2R in Alzheimer’s Disease: Synaptoprotection

Several lines of evidence connect S2R to Alzheimer’s disease; these include its localization to neuronal synapses, amyloid-β oligomers, and learning and memory (Figure 2, Table 1), including data from studies genetically or pharmacologically manipulating S2R constituents and interacting proteins, including PGRMC1. When amyloid-β oligomers bind to PrP^c^, long-term potentiation and the formation of new memories are disrupted [129,130,131]. Synaptic loss is a hallmark of Alzheimer’s disease [132] and other neurodegenerative diseases in which there is a significant, progressive reduction of synaptic density in disease-related brain regions. The loss in synapses occurs over the course of the disease as demonstrated in post-mortem studies, as well as in human positron emission tomography live imaging studies [133].

Long-term potentiation, thought to be a basis of learning and memory, is the classic experimental measure of synaptic plasticity [134]. S2R and PGRMC1 are linked to learning and memory through mechanism of action studies and efficacy studies in in vitro and in vivo preclinical models. Mechanistically, PGRMC1 has a role in positioning netrin receptors in the cell membrane to mediate axon guidance [11,105,106]. Netrin receptors, also called UNC-40/Deleted in Colorectal Cancer, have a role in spatial memory formation in adult mice [135], and are required for the establishment of long-term potentiation [134], as well as mediating axon guidance [11,136]. While axonal guidance mechanisms are active in developing organisms and stabilize by adulthood, axonal guidance cues are “reactivated” in adults and are important in establishing long-term potentiation in the adult organism [134]. Netrin-1 binding results in increased glutamate (AMPA subtype) receptors being positioned in the cell membrane, the mechanism underlying the potentiated synaptic signal [134]. PGRMC1 also promotes membrane trafficking, another molecular underpinning required for the establishment of long-term potentiation [11,137]. PGRMC1 is a heme binding protein [103,138] and regulates carbon monoxide through oxidative heme degradation [139]. Carbon monoxide is required for the establishment of long-term potentiation [140]. S2R modulators have been shown to rescue learning and memory in Alzheimer’s mouse models [2,64].

### 6.2. Rationale for S2R Modulators for Alzheimer’s Disease: Synaptoprotective, Restoration of Function

**Figure 4:**
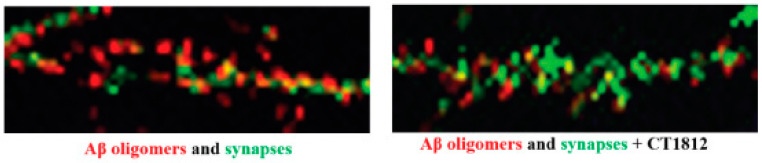
S2R modulator CT1812 slows the loss of synapses that is triggered by amyloid-β oligomers in vitro. High-resolution images of primary neuronal cell cultures exposed to β-amyloid oligomer are shown before the addition of CT1812 (**left**) and after the addition of CT1812 (**right**). Amyloid-β oligomers (red) bind to synaptic receptors and reduce numbers of synapses (Drebrin, green). The addition of CT1812 displaces amyloid-β oligomer binding and appears to block the effects induced by the amyloid-β oligomers, with the synapse numbers remaining at levels similar to control cultures. Reprinted with permission from Ref. [64], 2021, Izzo, et al.

**Figure 5:**
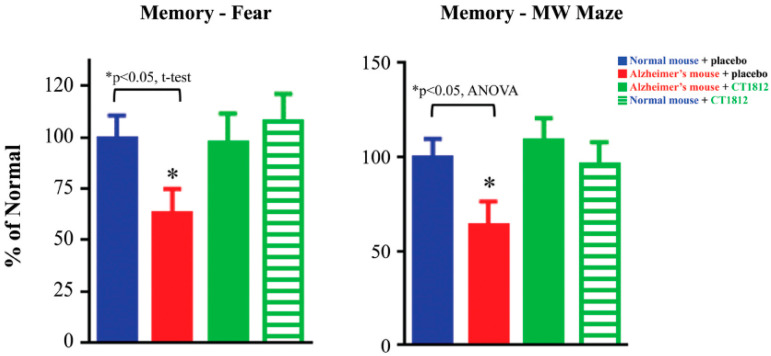
S2R modulator CT1812 restores functional capabilities in a mouse model of Alzheimer’s disease. Transgenic mice exhibiting phenotypes associated with Alzheimer’s disease (red bars) performed significantly worse in both the fear trigger recall test and Morris water (MW) maze tests when compared with normal, non-transgenic mice (blue bars). After administration of CT1812, however, the Alzheimer model mice (green bars) performed similar to that of wild-type mice. These results are illustrative of the restorative effects of CT1812 on synaptic proteins and the animal’s functional capabilities. Adapted from Ref. [64], 2021, Izzo, et al.

S2R modulators tested by several independent groups have been shown to be neuroprotective, be synaptoprotective, block toxic amyloid-β oligomers from binding to neuronal synapses, and restore neuronal functioning and cognitive deficits across various preclinical models, from neurons in culture to in vivo Alzheimer’s disease models from *C. elegans* to rodents [141]. Neuroprotection was demonstrated with two S2R-binding norbenzomorphan analogues (SFM-1500 and SAS-1121) in a *C. elegans* model of amyloid precursor protein-induced progressive neurodegeneration [142]. These S2R modulators reduced neurodegeneration in mutant *C. elegans* models to wild-type control levels [142]. In zebrafish, several S2R modulators with polypharmacology, exhibiting activity on multiple targets, demonstrated synaptoprotection in two stress-induced models of synaptic degeneration in zebrafish [83]. Four compounds were identified that are anti-acetylcholinesterases and glutamate receptor (NMDA-GluN2B subtype) ligands, one of which is a 2-(diisopropylamino)ethyl derivative that also binds S2R with high affinity and with seven-fold greater selectivity over the sigma-1 receptor [83]. This compound exhibited higher protective effects from acute amyloidosis-induced synaptic degeneration compared with donepezil, which is an acetylcholinesterase inhibitor not an S2R ligand [83]. The use of selective S2R modulators in zebrafish is warranted to confirm if this synaptoprotective effect observed is partially or wholly mediated by S2R activity. In a nerve-growth-factor-treated neurite outgrowth assay in PC12 cells, treatment with the S2R modulator PB28 enhanced nerve-growth-factor-induced neurite outgrowth. However, it should be noted that other S2R modulators exhibited the inverse phenotype; albeit, this was only tested at a high concentration (10 µM) [143]. Further studies on primary neurons or human induced pluripotent stem cell-derived neurons are warranted to determine if this effect is specific to PC12 cells or extends to primary neurons and more physiologically relevant neuronal cell culture models. In primary rat neurons, S2R modulators by CT0093, CT0109, CT1344, CT1346, and CT1812 demonstrate synaptic protection (Figure 4) and restoration of neuronal function [2,64]. In vivo, the S2R modulator SAS-0132 has been shown to produce neuroprotective, cognitive enhancing, and anti-inflammatory effects in a rodent model of Alzheimer’s disease [141]. Independently, several S2R modulators (CT0093, CT0109, CT1344, CT1346, and CT1812) rescued memory and cognitive deficits in an Alzheimer’s mouse model [2,64] (Figure 5). This suggests that one of the major debilitating symptoms of Alzheimer’s disease—the loss of ability to form new long-term memories—may be restored at a fundamental level by therapeutics targeting S2R.

### 6.3. Evidence of Synaptoprotection through S2R Modulators Blocking Amyloid-β Oligomer Toxicity

As previously discussed, amyloid-β oligomers bind to synapses [144] and interfere with the normal process of neurotransmission, long-term potentiation, and thereby memory formation [145]. Progressive cortical neuronal death is one of the primary pathologies of Alzheimer’s disease [146]. Synaptic loss, however, is not necessarily permanent and synapses can be regained or formed again once amyloid-β oligomers are removed [64]. Sparing and/or recovery of synapses is expected to result in slowing of the progression of neurodegeneration observed in Alzheimer’s disease. Modulating the S2R and thereby oligomer binding to neuronal synapses offers a traditional pharmaceutical approach to address the toxicities of amyloid oligomers. S2R modulators have the potential to act directly at the synapse by blocking amyloid-β oligomer binding and thereby oligomer-related synaptotoxicity.

Downstream of internalization, amyloid-β oligomers cause neuronal oxidative stress and dysfunction of autophagic and lysosomal vesicles. The role of autophagic/lysosomal dysfunction in Alzheimer pathology is evidenced by differential expression of key regulators of the pathway in individuals with Alzheimer’s disease compared with control individuals [147]. Moreover, pathological proteins associated with familial Alzheimer’s disease are either autophagy substrates, such as amyloid-β and tau, or linked to autophagy regulation, such as presenilin and clusterin [148]. The accumulation of autophagic vacuoles has been observed in dystrophic neurites of Alzheimer brains using electron microscopy of post-mortem biopsies [149], suggesting that the autophagic pathway is indeed impaired in affected neurons. These human data are also supported by data from animal models of Alzheimer’s disease [150,151]. Notably, a recent study examining five Alzheimer mouse models demonstrated autophagy dysregulation compared with wildtype animals [152]. These Alzheimer mouse models exhibited lysosomal membrane permeabilization, cathepsin release and cell death, accompanied by microglial invasion. Given the roles of S2R in regulating autophagy, as demonstrated in other models, targeting S2R to reverse amyloid-β toxicity may be beneficial in the context of autophagic dysfunction in Alzheimer’s disease.

Moreover, TMEM97 expression is significantly increased by ~1.5-fold in Alzheimer’s disease and is localized to an increased proportion (~1.8-fold) of synapses in brain tissue from individuals with Alzheimer’s disease when compared with healthy controls [65], which suggests a potential compensatory response to Alzheimer-related synaptic loss [69]. The interaction of S2R with amyloid-β in synapses is reduced in a dose-dependent manner in the presence of the S2R modulator CT1812 [65]. This interaction assessed biophysically, in the postmortem brain from individuals with Alzheimer’s disease, corroborates in vitro and in vivo evidence of displacement of amyloid-β oligomers [64], representing a potential mechanism of action underlying the protection against amyloid-β oligomer-induced synaptic degradation [153,154,155,156,157] observed with S2R modulators [64]. In sum, results from studies in preclinical Alzheimer’s models support that targeting S2R with a small-molecule approach may be a beneficial therapeutic strategy by displacing synaptotoxic amyloid-β oligomers, and preventing synaptotoxicity, thereby enabling synapses to recover and slow cognitive decline [2,3].

### 6.4. Preclinical Evidence for CT1812, Currently in Clinical Development for Alzheimer’s Disease

CT1812 is an orally delivered small molecule that penetrates the blood–brain barrier and binds selectively to S2R that is currently under development by Cognition Therapeutics, and in Phase 2 clinical trials for Alzheimer’s disease (NCT03507790, NCT04735536, NCT05531656). CT1812 employs a novel and fundamentally distinct mechanism-of-action from most other Alzheimer’s disease therapeutics. CT1812 selectively prevents and displaces amyloid-β oligomers from binding to neuronal synapses, thereby mitigating downstream toxicity. In preclinical studies, CT1812 also slows amyloid-β oligomer-induced loss of synapses and restores synaptic activity (Figure 4) [64], which may reverse downstream alterations related to membrane trafficking.

Interestingly, CT1812 phenocopies the protective A673T mutation in amyloid precursor protein, in that CT1812 reduces the binding of toxic β-amyloid oligomers to synapses, just as the A673T mutation amyloid-β oligomers demonstrate a four-fold reduction in binding compared with wild-type amyloid precursor protein [158]. This is notable as therapeutic approaches emulating phenotypes of protective genetic risk factors may increase the probability of success in the clinic.

The protective benefits of CT1812 observed in in vitro assays are supported by functional in vivo assessments of CT1812 [64]. In APPswe/PS1dE9 transgenic mice, an Alzheimer’s disease mouse model, the memory was tested based on the animal’s ability to recall fear-inducing triggers, and its performance in the Morris water maze. In both assays, the transgenic mice exhibited significant cognitive deficits that were prevented with CT1812 treatment (Figure 5).

**Figure 6:**
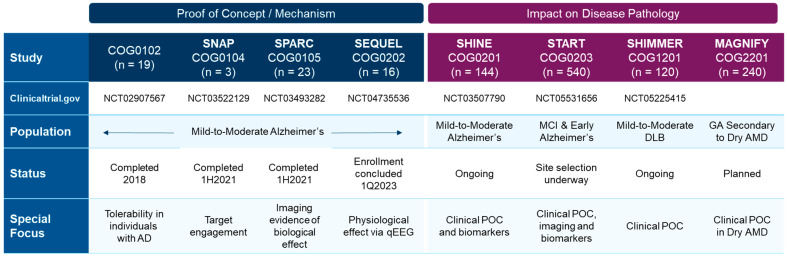
Completed and ongoing or planned clinical trials with the S2R modulator CT1812 in Alzheimer’s disease, dementia with Lewy bodies (DLB), and dry AMD patients.

These preclinical results have begun to be validated in completed clinical trials assessing safety and pharmacokinetic and pharmacodynamic outcomes [64,159], and will continue to be evaluated in ongoing Phase 2 clinical trials assessing efficacy (Figure 6).

### 6.5. Rationale for Targeting S2R for α-Synucleinopathies

A body of evidence exists tying S2Rs to α-syncleinopathies. α-Synuclein is a protein primarily found in neural tissue that has a role in neurotransmission. In α-synucleinopathies such as dementia with Lewy bodies and Parkinson’s disease, α-synuclein builds up in the brain and forms oligomers that saturably bind to neurons where they impair critical cellular processes, causing synaptic dysfunction and eventual loss. As with amyloid-β oligomers in Alzheimer’s disease, α-synuclein oligomers are highly toxic when bound to brain cells and internalized [160]. α-Synuclein oligomers are known to induce cellular stress responses, including aberrant autophagy-lysosome pathways [161,162], dysregulation of lipid metabolism [163,164], and a reduction in membrane trafficking [4]. More specifically, neuronal pathology observed in α-synucleinopathies includes deficits in membrane trafficking and an upregulation in lysosomal-associated membrane protein-2A, a protein critically required for chaperone-mediated autophagy [4]. These effects were observed with both synthesized α-synuclein oligomers and α-synuclein oligomers derived from brain tissue from individuals with Parkinson’s disease. S2R in close association with PGRMC1 regulates cell pathways known to be impaired in α-synucleinopathies, such as autophagy, vesicle trafficking, and lipid synthesis. Similar to amyloid-β oligomers, α-synuclein oligomers interact with PrP^c^ [42]. α-Synuclein interacts with PrP^c^ to induce cognitive impairment through metabotropic glutamate receptors, and blockage of these glutamate receptor-mediated signaling events restores cognitive deficits [42]. Given that PGRMC1 and S2R can regulate proteins and processes underlying α-synuclein oligomer toxicity, compounds that bind to S2R and block α-synuclein binding and/or internalization are expected to be disease-modifying [4].

### 6.6. Small Molecules Targeting S2R for α-Synucleinopathies

**Figure 7:**
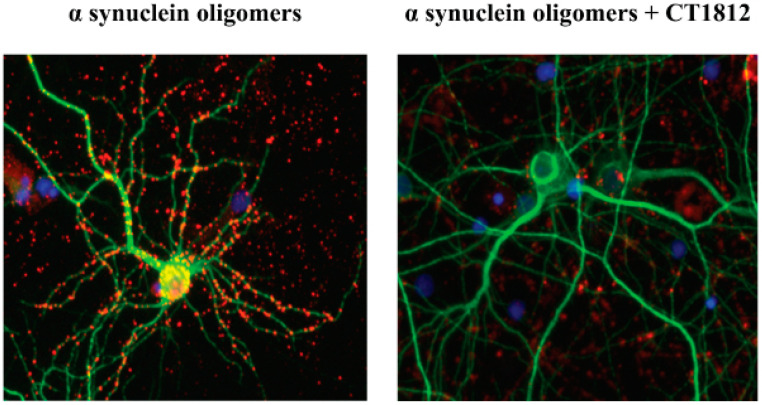
S2R modulators block the effects of α-synuclein oligomer binding to neurons. Representative microscopy images of primary neuronal cell cultures exposed to α-synuclein oligomer are shown with vehicle treatment (**left**) or after the addition of an S2R modulator (**right**). α-Synuclein oligomers (red) bind to neuronal processes (MAP2, green). The addition of an S2R modulator decreases the levels of α-synuclein oligomers bound to neurons [4].

The results of in vitro studies suggest that S2R modulators may have disease modifying effects on α-synucleinopathies by reversing pathway disruption and dysregulation caused by α-synuclein oligomers. α-Synuclein oligomers were found to bind to neurons in culture and are internalized (Figure 7 [4]). With the addition of an S2R modulator, the binding and thus internalization of the α-synuclein oligomers are inhibited (Figure 7 [4]).

**Figure 8:**
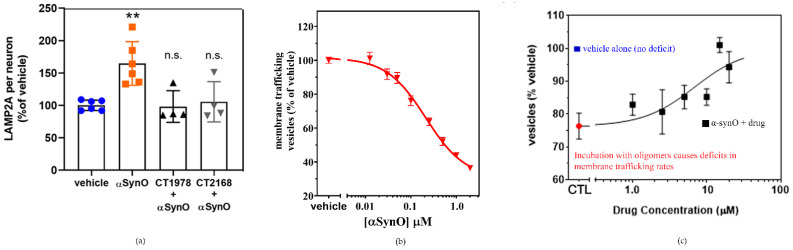
S2R modulators reverse the effects of α-synuclein oligomers on lysosomal-associated membrane protein 2A expression and trafficking. (**a**) Microscopy images of a cell culture exposed to α-synuclein oligomer with or without S2R modulators and stained for lysosomal-associated membrane protein 2A (LAMP2A) were quantified. (** *p* < 0.01, ANOVA with Dunnett’s test for multiple comparisons; n.s., not significantly different compared with vehicle-treated cells.) (**b**) Cultures treated with increasing concentrations of α-synuclein oligomer (α-synO) exhibit membrane trafficking deficits. (**c**) These trafficking deficits are blocked by treatment with S2R modulators. Adapted from Ref. [4], 2021, Limegrover, et al.

The potential for S2R modulators to reverse the deleterious cellular effects of α-synuclein oligomers is also reflected in the in vitro analysis of lysosomal-associated membrane protein 2A. Lysosomal-associated membrane protein 2A is a critical component of chaperone-mediated autophagy, one of several processes that eliminate damaged cellular proteins. Its expression is upregulated in the presence of the toxic α-synuclein oligomer (Figure 8a [4]), likely a compensatory mechanism in response to the cellular insult. Wild-type α-synuclein has been shown to be selectively degraded via chaperone-mediated autophagy [165]. Pathogenic α-synuclein mutants bind to lysosomal-associated membrane protein 2A and act as uptake blockers, inhibiting overall chaperone-mediated-autophagy-mediated degradation. Moreover, accumulation of soluble α-synuclein oligomers inhibits proteasomal function [166], which in turn can activate compensatory autophagic pathways. S2R modulators that block membrane trafficking deficits caused by α-synuclein oligomers are observed to inhibit the upregulation of lysosomal-associated membrane protein 2A [4]. As these ligands are selective for S2R, their ability to reverse the effects of α-synuclein on lysosomal-associated membrane protein 2A expression provides compelling evidence of the importance of S2R in the regulation of protein degradation pathways. In vitro analysis further illustrates α-synuclein oligomer-induced dose-dependent inhibition of membrane trafficking (Figure 8b, [4]). Importantly, α-synuclein oligomer-related inhibition was noted to be four-fold higher than that observed with high concentrations of monomeric α-synuclein, illustrative of the significantly greater toxicity of α-synuclein oligomers. The addition of a S2R modulator (Figure 8c) was observed to reverse the membrane trafficking deficit related to the presence of α-synuclein oligomer, while having no effect on membrane activity when dosed in its absence [4].

High-throughput screening of two small-molecule libraries identified several S2R-specific drug candidates for development as therapies for α-synucleinopathies, including Parkinson’s disease [4]. From the National Institutes of Health Clinical Collection libraries from the National Institutes of Health Small Molecule Repository, lovastatin and carbamazepine were identified as possible therapeutic candidates, blocking α-synuclein-induced, S2R-mediated, membrane-trafficking deficits at low concentrations, but these compounds were found to be neurotoxic at concentrations > 5 µM. S2R modulators from the Cognition Therapeutic small-molecule library were found to block α-synuclein-induced, S2R-mediated, membrane-trafficking deficits and upregulation of the critical chaperone-mediated autophagy protein lysosomal-associated membrane protein 2A with high affinity, without evidence of cytotoxicity at higher concentrations [4].

Notably, up to 80% of individuals with dementia with Lewy bodies exhibit amyloid-β pathology as well as α-synuclein pathology [167]. Given the ability of S2R modulators to rescue both amyloid-β oligomer and α-synuclein oligomer deficits [2,3,4,64], there is promise that S2R may be beneficial in patients with dementia with Lewy bodies.

A Phase 2 randomized, double-blinded, placebo-controlled clinical trial, SHIMMER, (NCT05225415) testing the S2R modulator CT1812 in individuals with dementia with Lewy bodies is ongoing and was initiated in 2022 by Cognition Therapeutics (Figure 6). The study design includes three dose groups, two active treatment cohorts, and a placebo group, and will assess safety, pharmacodynamic, and efficacy endpoints including cognitive assessments including the Montreal Cognitive Assessment. Findings from this trial may help to clarify whether the hypothesis that S2R modulators are beneficial for individuals with α-synucleinopathies such as dementia with Lewy bodies is correct.

### 6.7. Rationale for S2R in Dry AMD

Several lines of evidence suggest that modulation of S2R may provide significant therapeutic utility for the treatment of dry AMD. First, human genetics point to S2R as a promising therapeutic target, as indicated via several large-scale, independent genome-wide association studies. These studies indicate that a single nucleotide polymorphism in the TMEM-VTN locus confers decreased risk for dry AMD [168,169]. It remains unknown if this mutation confers a change in S2R expression levels; however, animal models of dry AMD lend support for targeting S2R. S2R expression is elevated in retinal pigment epithelium-specific *Clic4* knockout mice, which exhibit both functional and pathological hallmarks of dry AMD. Conversely, *TMEM97* knockout mice have exhibited conflicting results in dry AMD models, which are likely dependent upon the type of stressor used to induce pathology. Intravenous administration of sodium iodate to *TMEM97* knockout animals induced greater reactive oxygen species accumulation and retinal pigment epithelium cell death than in similarly treated wildtype animals [119]. Other studies, however, demonstrate protective effects of decreased S2R. Either *TMEM97* knockout or the S2R-selective modulator DKR-1677 provided neuroprotection against ischemia-induced murine retinal ganglion cell degeneration [73]. Furthermore, using an illumination-induced degeneration model of AMD in Abca/Rdh8-null mice, S2R-selective modulation using CM398 reduced photoreceptor loss and accumulation of retinal autofluorescent granules, a marker of retinal pigment epithelium cell degeneration [84]. Taken together, these data indicate that S2R function is important in dry AMD pathogenesis and in other retinopathies. Available models of the disease are limited, however, because none of the current in vitro or in vivo models can faithfully recapitulate all the phenotypes or time-course of the pathology observed in humans. Thus, it will be important to further elucidate the mechanisms underlying dry AMD and that represented in various disease models to better predict how modulation of S2R may impact the disease.

Dry AMD remains a leading cause of blindness, and there are no approved therapies at present. Thus, S2R modulation may fill an important unmet therapeutic need. The proteomics data from cerebral spinal fluid and plasma were examined from a clinical trial of CT1812 in treatment of mild/moderate Alzheimer’s disease for 6 months (SHINE-A study), and the expression of many proteins known to be disrupted in dry AMD was differentially expressed with treatment with CT1812 compared with placebo [170]. This provides preliminary proof-of-principle that CT1812 may alter dry AMD-relevant protein expression in an aged human population. In vivo animal studies demonstrated that efficacious levels of CT1812 exist in the retina, as well as the brain, with oral therapeutic doses [64]. A clinical study directly testing the efficacy of CT1812 as treatment for dry AMD is planned (Figure 6).

### 6.8. Other Indications in Focus for S2R Therapeutic Intervention

Three additional diseases affecting the central nervous system warrant mention here; S2R has been implicated in Niemann–Pick disease type C, schizophrenia, and Huntington’s disease. Niemann–Pick disease type C is a progressive disease characterized by abnormal transport and storage of cholesterol and other lipids in cells. Individuals with Niemann–Pick disease type C exhibit a variety of visceral and neuropsychiatric symptoms, which include cognitive decline in adulthood [171,172,173]. S2R has been implicated in the process of abnormal cholesterol trafficking in Niemann–Pick disease type C, and data have demonstrated that knockdown of S2R expression increased intracellular Niemann–Pick disease type C 1 protein and restored LDL-cholesterol and lysobisphosphatidic acid transport [94]. This result was observed in a Niemann–Pick disease type C model developed in HeLa cells, and in five primary fibroblast cell lines from individuals with Niemann–Pick disease type C [94]. It is interesting that along with cognitive decline, retinal axonal degeneration has been recently described to be another manifestation of Niemann–Pick disease type C in younger individuals [174]. Therapeutics developed for other degenerative diseases associated with S2R may be effective also for retinal axonal degeneration observed in Niemann–Pick disease type C.

Roluperidone (MIN-101) is a non-selective S2R, serotonin, and adrenergic receptor small-molecule ligand that has been in development for the treatment of schizophrenia [175]. In a Phase 2b randomized controlled clinical trial, roluperidone exhibited promising therapeutic effects on cognitive function in individuals with schizophrenia [175]. It is not yet known if the mechanism underlying the cognitive benefits involves S2R, serotoninergic or adrenergic receptors, or a combination thereof.

S2R modulators have also been shown to be neuroprotective in neuronal culture models of Huntington’s disease [5]. The underlying cause of Huntington’s disease is well-known to be the expansion of a CAG repeat in the huntingtin gene. This results in changes in the interaction of the mutated huntingtin protein with multiple other proteins, affecting multiple cellular processes, including accumulation of the misfolded huntingtin protein and production of toxic huntingtin protein fragments [176,177]. Resulting cell death occurs via both excitotoxicity and apoptosis primarily in the cortex and striatum in the brain [178]. S2R, but not sigma-1 receptor modulators reduce the neuronal toxicity induced by Htt-N586-82Q in primary cortical neuronal cultures [5]. Two effects of the mutant huntingtin protein have been shown to be impaired vesicle trafficking and abnormal intracellular Ca^2+^ levels and signaling. These may represent links to the dose-dependent neuroprotective effects observed with several selective and high-affinity S2R ligands (AMA-1127, DKR-1051, UKH-1114, EES-1686, and BEA-1687) tested in murine primary cortical neurons transfected with the human mutant huntingtin protein [5]. It is also possible that the neuroprotective effects of the S2R ligands in the Huntington model are due to a reduction in binding of toxic huntingtin protein fragments, similar to that observed with displacement of toxic amyloid-β and α-synuclein protein fragments. Despite the evidence that these S2R ligands are protective for these conditions, no clinical trials are registered in ClinicalTrials.gov using the aforementioned molecules for either indication.

## 7. Conclusions

Alzheimer’s disease, α-synucleinopathies (Parkinson’s disease and dementia with Lewy bodies), and dry AMD are several age-related degenerative diseases of the central nervous system for which current available therapeutics are severely limited. The biological understanding of S2R has recently come into clarity, rapidly advancing the developments in the field and possible therapeutics targeting S2R. S2R (TMEM97) functions in close association with PGRMC1 and actuates its functions through the formation of protein–protein interactions, including a protein complex that binds oligomers and LDLR (Table 1). S2R is broadly expressed, but importantly is expressed in tissues relevant to age-related degenerative disorders of the central nervous system, including the brain and retina, and is intimately involved in regulating disease-relevant functions. These biological processes include regulation of autophagy, cholesterol homeostasis, membrane trafficking, and clearance of toxic amyloid-β and α-synuclein fragments from neurons. S2R regulates synaptic plasticity and synaptic density in Alzheimer’s disease, representing a fundamental mechanism underlying the devastating loss of memory and further cognitive decline in Alzheimer’s disease.

Small molecule therapeutics targeting S2R have exhibited increasing success in amelioration of pathology observed in Alzheimer’s disease, including restoration of membrane trafficking, synaptic plasticity, synaptic density, and memory ability, primarily via displacing the binding of toxic amyloid-β oligomers. This mechanism underlying synaptoprotection is likely similar for the neuroprotection and restoration of cognitive function observed with S2R modulators in α-synucleinopathies. S2R modulators also exhibit promising effects in models of dry AMD. The underlying mechanism is unknown at present but may involve restoration of function against toxic proteins and oxidative stress. Although S2R modulators are not in the clinic for other age-related degenerative diseases of the central nervous system, such as Huntington’s disease, evidence is in support of this as a potential future avenue worthy of pursuit. Given that TMEM97 is integral to regulation of Niemann–Pick disease type C1, this also paves the way to understanding further whether S2R modulators or genetic manipulation of *TMEM97* may be fruitful in ameliorating aspects of Niemann–Pick disease type C. As pharmacological agents directed at S2R are developed, it is likely that effective therapeutics for a host of indications will emerge, as several recent reviews discuss [87,179,180,181].

Future resolution of S2R via X-ray crystallography bound with multiple selective ligands or with protein binding partners in cell types of interest will provide valuable insight into the structural status of the proteins involved in response to the binding of each ligand/protein. The various protein arrangements (e.g., phosphorylation, dimerization, heme binding) of S2R and its closely associated proteins are not fully discovered, and the continued description of their interactions will likely illuminate the functional role of each and their role in pathology and disease.

While great strides towards better understanding S2R pharmacology, biology, and therapeutic potential have been made in recent years [4,10,54,62,64,182,183,184], a number of outstanding questions remain. What are the precise molecular mechanisms underlying the functional effects of S2R in neurons and other cell types described by the field? Are there cell type-specific molecular interactions that underlie these functions, and are there basic aspects common across cell types expressing S2R? A continued effort in the field will be critical to address these questions and paint a clear picture delineating S2R signaling and function. Lastly, and arguably most importantly, will the efficacy observed in preclinical studies for neurodegenerative conditions translate to the clinic? Understanding whether S2R modulators prevent or remedy the toxic effects of amyloid-β and α-synuclein oligomers, and protect synapses in individuals with Alzheimer’s disease or dementia with Lewy bodies will be important; however, ultimately, improvement in the quality of lives of individuals affected by these diseases will be of utmost value. We look forward to learning the outcome of clinical trials with the S2R modulator CT1812 in Alzheimer’s disease and dementia with Lewy bodies to determine if S2R modulation may provide clinical benefits for people with these devastating diseases.

## Figures and Tables

**Table 1:** Key functional roles of S2R and the molecular players involved.

Functions of S2R	Molecules Involved	Disease Relevance	Sections in Text
Blocks amyloid-β oligomers from binding neuronal synapses	TMEM97, PGRMC1 oligomer receptor	Alzheimer’s disease	5.4, 6.1–4
Blocks α-synuclein oligomers from binding neuronal synapses	TMEM97	Dementia with Lewy bodies Parkinson’s disease	6.5–6
Mediates synaptoprotection	TMEM97, PGRMC1 mGluR5, oligomer receptor	Alzheimer’s disease Parkinson’s disease Dementia with Lewy bodies	6.1–3
Regulates autophagy	TMEM97, PGRMC1 LAMP2A, MAP1LC3B	Dry AMD Dementia with Lewy bodies Parkinson’s disease	5.4, 6.7 6.5–6
Regulates cholesterol homeostasis	TMEM97, PGRMC1, LDLR, Apo-E, NPC1	Alzheimer’s disease Niemann–Pick disease type C	5.4, 6.1 5.4, 6.8
Regulates membrane trafficking	TMEM97, PGRMC1, LDLR	Alzheimer’s disease Parkinson’s disease Dementia with Lewy bodies	6.1–4 6.5–6

Abbreviations: AMD, age-related macular degeneration; Apo-E, apolipoprotein-E; LAMP2A, lysosomal-associated membrane protein 2A; LDLR, low-density lipoprotein receptor; MAP1LC3B, microtubule-associated proteins 1A/1B light chain 3; mGluR5, metabotropic glutamate receptor 5; NPC1, Niemann–Pick protein C1; PGRMC1, progesterone receptor membrane component 1; S2R, sigma-2 receptor; TMEM97, transmembrane protein 97.

## Data Availability

Not applicable.
